# Effects of Hydrolysable Tannin with or without Condensed Tannin on Alfalfa Silage Fermentation Characteristics and In Vitro Ruminal Methane Production, Fermentation Patterns, and Microbiota

**DOI:** 10.3390/ani11071967

**Published:** 2021-06-30

**Authors:** Lei Chen, Xueyan Bao, Gang Guo, Wenjie Huo, Qingfang Xu, Cong Wang, Qinghong Li, Qiang Liu

**Affiliations:** 1 College of Animal Science, Shanxi Agricultural University, Taigu 030801, China; CL1016ZJ@126.com (L.C.); 18235417674@163.com (X.B.); guosteel1984@163.com (G.G.); huohuo-1982@163.com (W.H.); wangdx0321@163.com (C.W.); sxaulqh88@126.com (Q.L.); 2 College of Grassland Science, Shanxi Agricultural University, Taigu 030801, China; xuqfsxau@126.com

**Keywords:** methane mitigation, proteolysis, ruminal fermentation, ruminal microbiota, silage, tannins

## Abstract

**Simple Summary:**

The sustainability of livestock husbandry requires efficient nitrogen and energy utilization by ruminants fed high-forage diets. The objective of this study was to evaluate the effect of hydrolysable tannin (HT) without or with condensed tannin (CT) on modulating the ensiling characteristics, methane production, ruminal fermentation profile, and microbiota of alfalfa silage. The results showed that adding HT, alone or in combination with CT, to alfalfa at ensiling improves fermentation quality and reduces ruminal methane production of alfalfa silage. Moreover, HT and CT in combination are more potent in modulating fermentation quality and methanogenesis than HT only; however, the high level of inclusion will impair silage degradation and microbiota in the rumen. Importantly, the results from this study revealed that a combination of HT and CT with complementary mechanisms at low doses can improve N utilization efficiency and methane mitigation of silage feed without adverse effects on ruminal fermentation patterns and microbiota. The findings in this study are of practical importance for the effective use of tannins as an additive for improving silage quality and utilization by ruminants.

**Abstract:**

This study was conducted to evaluate the potential of hydrolysable tannin (chestnut tannin, CHT) without or with condensed tannin (quebracho tannin, QT) for modulating alfalfa silage fermentation characteristics and in vitro ruminal methane (CH_4_) production, fermentation profile, and microbiota. Alfalfa (235 g/kg fresh weight) was ensiled with no tannins (control), 2% CHT (CHT2), 5% CHT (CHT5), the combination of CHT and QT at 1% each (CHQ2), and CHT and QT at 2.5% each (CHQ5) of forage dry matter (DM). The CHQ2 treatment was more effective in reducing DM losses, pH, and ammonia–nitrogen to total nitrogen ratios of alfalfa silage than CHT2 and CHT5 treatments. All tannin treatments decreased ruminal CH_4_ production, and the magnitude of the decrease was greater for the combinations than the individual ones. Total volatile fatty acid (VFA) concentrations and DM degradation decreased by tannin treatments, but microbial protein (MCP) synthesis increased. The total VFA concentrations and DM degradation were lower with CHQ2 treatment than with CHT5 and CHQ5 treatments, but the MCP concentrations were comparable among these treatments. Tannin inclusion decreased the abundance of the anaerobic fungi *Ruminococcus albus* and *Ruminococcus flavefaciens*, but enhanced *Fibrobacter succinogenes*. The combination of CHT and QT alleviated the inhibition of CHT supply alone in *Butyrivibrio fibrisolvens*, *Ruminobacer amylophilus*, and *Prevotella ruminicola* as well as protease. The results revealed that a combination of HT from CHT and CT from QT at a low level can reduce proteolysis and CH_4_ production of alfalfa silage without impairing ruminal fermentation and microbiota.

## 1. Introduction

The livestock husbandry has long been the pillar industry of agriculture and rural economy in most developing and developed countries. Ruminants, as an important subsector of the livestock production system, have great significance to humans since they can utilize fibrous plants and by-products that are indigestible for humans to produce high-protein foods such as milk and meat [1]. However, ruminant production is accompanied by enteric methane (CH_4_) emission in large quantities, leading to environmental impact. Methane production from ruminants accounts for 16–25% of the global greenhouse gas emission and about 33% of global anthropogenic CH_4_ emission. The CH_4_ emission also represents a loss of 2% to 15% of the ingested energy [2]. Besides contributing to CH_4_ emission, ruminants have low dietary nitrogen utilization efficiency and excrete 75% to 95% of the nitrogen intake, particularly for forage diets high in soluble protein [3,4]. Ultimately the excreted nitrogen exists in the form of ammonia–nitrogen (NH_3_-N) and nitrous oxide that can cause air and groundwater pollution. In upcoming decades, the global consumption level of beef and milk will continue to rise with increasing human population; meanwhile, these ensuing environmental problems would be increasingly prominent. Therefore, sustainable mitigation approaches for CH_4_ emission and nitrogen excretion are needed to be developed to improve forage conversion efficiency and alleviate the environmental impact of ruminant production.

One such promising mitigation approach is the incorporation of tannins in forage. Tannins are the second-most widespread phenolic compounds in the plant kingdom and are traditionally classified into hydrolysable tannins (HT) and condensed tannins (CT) [5]. Tannins are regarded as natural ruminant feed additives that can modulate protein metabolism [6], enteric CH_4_ emission, and animal performance [7]. To date, HT and CT have been repeatedly evaluated, mostly individually, for their efficiency to prevent proteolysis during ensiling and rumen fermentation or to reduce enteric methane production from ruminants [8,9,10]. However, HT and CT often exhibit negative effects on feed digestion and rumen fermentation when applied at levels high enough to obtain a desirable reduction in proteolysis and CH_4_ production, while they lead to little inhibition to proteolysis and methane production when applied at low levels that hardly affect feed digestion or rumen fermentation. It has been reported that HT appears to reduce proteolysis and methane production more by inhibiting functional rumen microbes, while CT acts more by reducing protein and carbohydrate degradation with its molecules binding capacity [4,11]. Thus, we hypothesized that a combination of HT and CT may be more effective in modulating proteolysis and methane mitigation with complementary mechanisms, achieving the desirable reduction of proteolysis and CH_4_ production at low levels without adverse effects on forage digestion or fermentation.

Alfalfa is a principal source of home-grown protein on farms and is widely used as forage for grazing cattle and dairy cows [12]. However, the protein utilization efficiency in ruminants fed alfalfa silage was normally low due to extensive proteolysis of alfalfa silage, especially natural alfalfa silage, resulting in an increase in ruminal NH_3_-N concentration (an indicator for N excretion). Therefore, the objectives of this study were to evaluate the effects of HT alone and in combination with CT at low and high levels on fermentation characteristics of high-moisture alfalfa silage and their effects on ruminal CH_4_ production, nutrient degradation, fermentation, enzyme activity, and microbiota.

## 2. Materials and Methods

### 2.1. Forage, Treatments, and Ensiling

The first-cut of alfalfa was harvested at the 10% bloom stage from 3 random locations in the experimental field of Shanxi Agricultural University, Taigu, China (37°43′ N, 112°55′ E). Alfalfa was chopped with a paper cutter to about 20 mm lengths and placed on a polyethylene sheet. Five replicated piles of forage (1.5 kg per pile) were prepared from each of the 3 locations to produce 15 total piles. The 5 piles in each location for experimental treatments were reserved in a cool sunshade area for a short time before ensiling. During this time, one sample from each location was obtained for chemical composition and microbial analysis of fresh forage. The chemical composition and microbial counts of fresh alfalfa are detailed in Table 1.

The HT extract (chestnut tannin, CHT; 92% tannin; GEEKEE Biotech Co., Ltd., Xi’an, China) and CT extract (quebracho tannin, QT; 99% tannin; GEEKEE Biotech Co., Ltd., Xi’an, China) were kept in fine dry powder. One replicated pile from the 5 piles in each location was separately assigned to one of the following experimental treatments: no tannins (control), CHT at 20 g kg^−1^ DM (low level; CHT2), CHT at 50 g kg^−1^ DM (high level; CHT5), the combination of CHT and QT at 10 g kg^−1^ DM each (low level; CHQ2), and the combination of CHT and QT at 25 g kg^−1^ DM each (high level; CHQ5). All tannin treatments were dissolved in distilled water and sprayed uniformly onto the chopped alfalfa (10 mL kg^−1^ fresh weight), and an equal dose of distilled water was applied to the control. About 1 kg of treated forages was packed manually into a pre-weighed polyethylene plastic bag (26 × 36 cm) equipped with a one-way valve to allow gas escape. The filled bags were vacuum-sealed using a vacuum machine (YMX-958-6L, Yiminxin Co., Ltd., Quanzhou, China), weighed, and stored for 60 d at room temperature (25−28 °C). After 60 d of ensiling, these bags were weighed before opening to estimate DM loss (DML), and then silages were sampled for chemical and microbial analyses, as well as in vitro incubation.

### 2.2. Chemical and Microbial Analyses

Water extract was obtained from fresh and ensiled alfalfa by macerating samples (20 g) with deionized water (60 mL) for 24 h at 4 °C, and then filtered to measure pH, buffer capacity (BC), water-soluble carbohydrates (WSC), NH_3_-N, and organic acids (lactic, acetic, and butyric acids). The pH was measured using an electrode pH meter (FE28, Mettler-Toledo Instruments Co., Ltd., Shanghai, China). The BC and WSC were determined with the method of Cavallarin et al. [13]. The NH_3_-N was measured by the method of phenolhypochlorite colorimetry, and organic acids were determined with high-performance liquid chromatography (HPLC) according to Chen et al. [14]. Fresh or ensiled alfalfa samples (10 g) were blended with 90 mL of sterilized saline solution (NaCl, 8.5 g/L) for 10 min, and the liquid was serially diluted 10-fold. The diluted samples were used to measure microbial counts by the plate count method. Lactic acid bacteria (LAB) and yeasts were incubated and enumerated using de Man, Rogosa, Sharpe (MRS) agar at 30 °C for 48 h and malt extract agar at 32 °C for 72 h, respectively. The microbial data were presented in the form of log10 on a fresh matter basis.

Fresh or ensiled alfalfa samples (500 g) were freeze-dried and ground to pass a 1 mm screen with a laboratory knife mill (FW100, Taisite instrument Co., Ltd., Tianjin, China) for nutrients analysis. Ground samples were analyzed for DM (934.01) and total nitrogen (TN; 984.13) based on the methods of AOAC [15]. Crude protein (CP) was calculated by multiplying TN by 6.25. Neutral detergent fiber (NDF; with heat stable α-amylase and sodium sulfite) and acid detergent fiber (ADF) were sequentially measured according to Van Soest et al. [16]. For determining proteolysis during ensiling, CP was divided into five fractions (PA, PB_1_, PB_2_, PB_3_, and PC) through degradable characteristics according to Cornell Net Carbohydrate and Protein System (CNCPS) [17]. Briefly, PA represents non-protein nitrogen (NPN), PB_1_ represents rapid degradation of true protein, PB_2_ represents intermediate degradation of true protein, PB_3_ represents slow degradation of true protein, and PC represents undegradable protein. The five protein fractions were analyzed with standardization procedures as described by Licitra et al. [18].

### 2.3. In Vitro Incubation

Rumen fluid was collected immediately before the morning feeding from three rumen fistulated cattle fed 60% corn silage and 40% concentrate at 0700 and 1800 h. The rumen fluid collected was homogenously mixed and strained through a sterilized muslin cloth (pore size 250 μm) into an O_2_-free thermos flask for use as inoculum. The substrate (0.5 g of freeze-dried silage) was weighed into a pre-weighed nylon bag (pore size 38–40 μm; Beijing First Beef Cattle Infor & Tech Research Center, Beijing, China), heat sealed and placed into a 100 mL serum bottle. Subsequently, the inoculation medium (60 mL), prepared aerobically with rumen inoculum (20 mL) and mineral buffer (40 mL) of Menke et al. [19], was dispensed into the serum bottles flushed with CO_2_. These bottles were closed with rubber stoppers and incubated in a water bath shaker at 39 °C for 48 h. Three independent incubation runs were conducted in three weeks. In each run, 15 sample bottles (5 treatments × 3 replicates) and 3 bottles as blanks (inoculation medium + empty nylon bags) were prepared.

The total gas volume was recorded every 6 h of incubation with the pressure transducer technique and corrected with blank bottles. After measuring gas volume, the gas produced of each bottle was collected with a gas-sampling bag (Hede Technologies Co., Ltd., Dalian, China) for later CH_4_ analysis. After 48 h of incubation, the pH of the incubation liquid was immediately measured with the electrode pH meter. Thereafter, all nylon bags were retrieved from the bottles, gently squeezed by hand, and the squeezed liquid from each bag was transferred to the corresponding incubation liquid. The nylon bags were washed with running water until the water became clear and were dried at 55 °C for 60 h. Differences in the amounts of DM, CP, and NDF between the substrates and the undegraded residues in the nylon bags were regarded as nutrient degradation. The bottles with the incubation liquid were fully stirred, and the incubation liquid was sampled. The incubation liquid sample (2 mL) was kept in a centrifuge tube at −80 °C for DNA extraction. The incubation liquid sample (10 mL) was centrifuged (12,000× *g*, 10 min, 4 °C), and the supernatant was kept at −20 °C for ruminal NH_3_-N and volatile fatty acid (VFA) analyses. The incubation liquid (5 mL) was used to determine microbial protein (MCP) using the Coomassie brilliant blue method as described by Pang et al. [20]. The CH_4_ concentrations of gas samples were measured with the method of carbon dioxide absorption as described by Fievez et al. [21].

### 2.4. Total DNA Extraction and Real-Time PCR

The incubation fluid samples (2 mL) from each bottle in each run were thawed at 4 °C, pooled, and blended well before DNA extraction. Then DNA was extracted using the TIANamp stool DNA isolation kit (Tiangen Biotech Co., Ltd., Beijing, China) with the repeated bead-beating method. The quantity and quality of extracted DNA were determined with a NanoDrop 2000 spectrophotometer (Thermo Scientific, Wilmington, DE, USA), and similar DNA concentrations across samples were obtained by adjusting the volume of samples [22].

Primer sets used for amplifying 16S rRNA genes of eubacteria and archaea, and the 18S rRNA gene of protozoa was commercially synthesized (BGI Life Tech Co., Ltd., Beijing, China; Supplementary Table S1). Primer-BLAST search of GenBank sequences was used to check the specificities of these primers. Regular PCR was used to generate sample-derived DNA standards for each qPCR assay. The procedure of amplification and specificity detection of PCR products was performed according to Du et al. [23]. The amplicons were purified using the MiniBest DNA Fragment Purification kit (Takara Biotechnology Co., Ltd., Dalian, China) and quantified using a spectrophotometer. The purified and quantified PCR products were serially diluted 10-fold with nuclease-free water to establish standard curves for targeted microbes.

The qPCR assay was performed using a StepOne Plus™ real-time PCR system (Applied Biosystems, Foster City, CA, USA). Each amplification reaction was done in duplicate with a 20 μL reaction mixture that contained 10 μL of Fast SYBR Green Mastermix, 0.4 μL ROX Reference Dye (50×), 2 μL of DNA template, 6.0 μL nuclease-free H_2_O, and 0.8 μL of each primer (10 μmol μL^−1^). The amplification condition included an initial denaturation step at 95 °C for 10 min, followed by 40 cycles of 95 °C for 15 s, optimal annealing temperature (Supplementary Table S1) for 1 min, and an elongation at 72 °C for 30 s.

### 2.5. Statistical Analyses

All statistical analyses were performed using SAS 9.2 (SAS Institute Inc., Cary, NC, USA) in a completely randomized design. The analysis of variance (ANOVA) was conducted using the GLM procedure of SAS 9.2. In the in vitro fermentation experiment, each incubation run represented the experimental unit. Data of the replicates of the three runs within the same sample of the substrate were averaged before statistical analysis. The model of ANOVA was as follows:

Y*_i_* = µ + α*_i_*+ ε*_i_*

where Y*_i_* is the dependent variable, µ is the least-square mean, α*_i_* is the tannin treatment effect, and ε*_i_* is the experimental error. Multiple comparisons between least-square means were conducted with Tukey’s test, and statistical significance was declared at *p* < 0.05.

## 3. Results

### 3.1. Ensiling Characteristics

**Figure 1:**
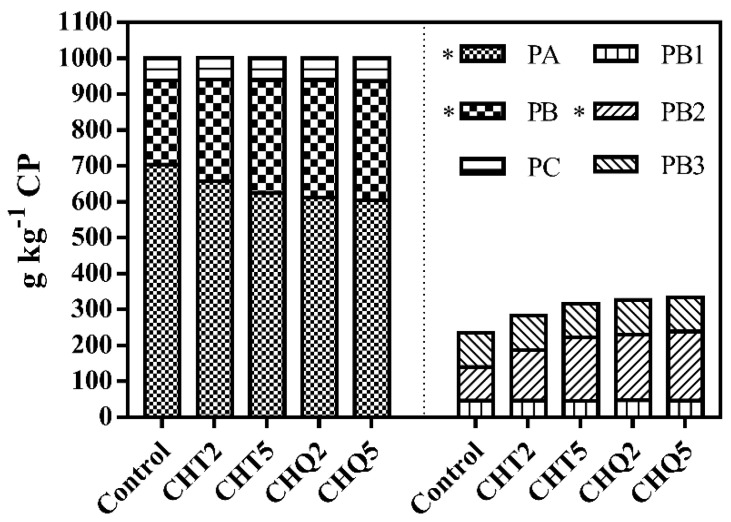
The effects of tannin treatments on protein fractions of alfalfa silage. Control = no additives; CHT2 = 20 g kg^−1^ DM of chestnut tannin; CHT5 = 50 g kg^−1^ DM of chestnut tannin; CHQ2 = 10 g kg^−1^ DM of gallnut tannin + 10 g kg^−1^ DM of quebracho tannin; CHQ5 = 25 g kg^−1^ DM of gallnut tannin + 25 g kg^−1^ DM of quebracho tannin. PA = non-protein nitrogen; PB = true protein; PB_1_ = rapid degradation of true protein; PB_2_ = intermediate degradation of true protein; PB_3_ = slow degradation of true protein; PC = undegradable protein. * represents significant difference at *p* < 0.05.

Compared with the control, all tannin treatments decreased (*p* < 0.001) DML, pH, acetic acid and butyric acid concentrations, NH_3_-N to TN ratios, and LAB counts, while increased DM (*p* < 0.001) and NDF (*p* = 0.004) concentrations (Table 2). The decreased and increased magnitudes of these detected variables were greater for combinations of CHT and QT than CHT alone except DM and NDF. Moreover, all tannin treatments decreased (*p* < 0.001) lactic acid concentrations except CHQ2. All tannin treatments affected (*p* < 0.001) PA, PB, and PB_2_ proportions but had no effects on PC, PB_1_, and PB_3_ proportions (Figure 1). Compared with the control, addition of tannins decreased (*p* < 0.001) the PA proportions, while increased (*p* < 0.001) the PB and PB_2_ proportions. The proportion of PA was lower (*p* = 0.002 for CHT5, *p* < 0.001 for CHQ2 and CHQ5), but the proportion of PB (*p* = 0.002 for CHT5, *p* < 0.001 for CHQ2 and CHQ5) and PB_2_ (*p* < 0.001 for CHT5, CHQ2, and CHQ5) was greater in silages produced using CHT5, CHQ2, and CHQ5 than CHT2.

### 3.2. Ruminal Gas and CH_4_ Production

**Figure 2:**
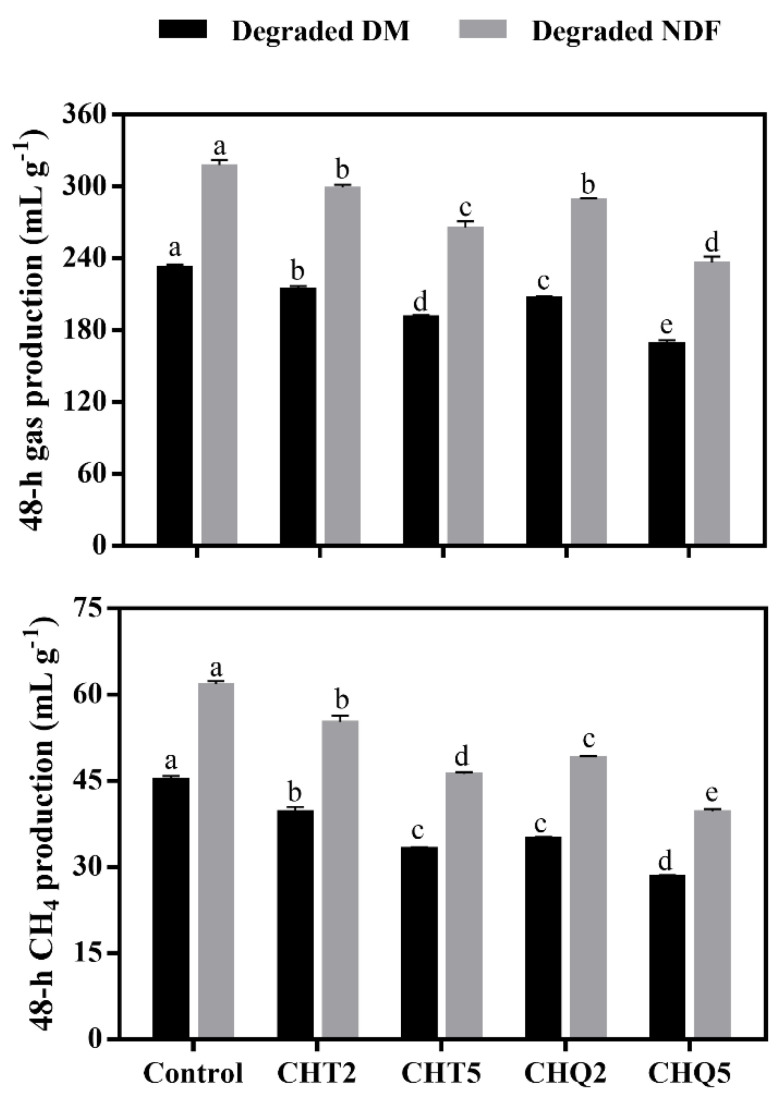
Gas and methane production in the in vitro fermentation cultures receiving untreated or tannin-treated alfalfa silage after 48 h incubation. Control = no tannins; CHT2 = 20 g kg^−1^ DM of chestnut tannin; CHT5 = 50 g kg^−1^ DM of chestnut tannin; CHQ2 = 10 g kg^−1^ DM of chestnut tannin + 10 g kg^−1^ DM of quebracho tannin; CHQ5 = 25 g kg^−1^ DM of chestnut tannin + 25 g kg^−1^ DM of quebracho tannin. Error bars represent the standard error of the means (*n* = 3), and different letters (a–e) above the same color column were significant (*p* < 0.05).

All gas and CH_4_ variables, the formation of per gram of degraded DM and NDF after 48 h incubation, are presented in Figure 2, respectively. Tannins, irrespective of type and level, had an inhibitory effect on gas and CH_4_ production, and the inhibitory effect was more evident for CHT5 and CHQ5 treatments. The combination of CHT and QT further decreased CH_4_ production compared with CHT alone, indicated by the averaged magnitude of CH_4_ reduction per gram of degraded DM (29.9% vs. 19.5%) and NDF (28.2% vs. 18.0%) compared with the control.

### 3.3. Ruminal Fermentation Patterns and Nutrient Degradation

The CHT alone or in combinations with QT at both levels did not affect the pH of the incubation fluid (Table 3). Total VFA concentrations were lowered (*p* < 0.001) by all tannin treatments compared with the control. Tannin treatments affected individual VFA except acetic and butyric acids. The propionic acid molar proportions increased (*p* < 0.001) for tannin treatments, and thus, the acetic acid to propionic acid ratio decreased (*p* < 0.001). The maximum alteration occurred with CHQ5 treatment in which propionic acid proportion increased by 15.9% compared with the control. Iso-butyric, iso-valeric, and valeric acids molar proportions were lower (*p* < 0.001) for all tannin treatments. The concentration of NH_3_-N decreased (*p* < 0.001) with CHT2 and CHT5 treatments compared with the control, and QT inclusion at the high level further decreased this variable. The concentration of MCP was increased (*p* < 0.05) by tannin treatments, and CHT5, CHQ2, and CHQ5 treatments had greater (*p* < 0.001) MCP concentrations than CHT2 treatments. The degradation of DM, CP, and NDF were lowered (*p* < 0.001) by all tannin treatments compared with the control, and CHQ5 treatment led to the lowest values.

### 3.4. Ruminal Enzyme Activity and Microbes

The activity of carboxymethyl-cellulase was lower (*p* = 0.047, *p* = 0.029) for CHT5 and CHQ5 treatments than the control (Table 4). The activity of α-amylase was lower (*p* = 0.008, *p* < 0.001) for CHT2 and CHT5 treatments than the control. All tannin treatments reduced (*p* < 0.05) the activity of cellobiase, xylanase, pectinase, and protease, and CHT5 treatment resulted in the lowest values.

The abundance of total methanogens and total anaerobic fungi was lower (*p* < 0.001) for all tannin treatments than the control. Total protozoa numbers for CHT2 and CHQ2 treatments were comparable to that for the control but were lowered (*p* < 0.001) to a great extent for CHT5 and CHQ5 treatments. Tannin treatments did not affect the abundance of total bacteria; however, the bacterial groups studied differed across treatments. Compared with the control, the abundance of the cellulolytic bacteria *Rumincoccus albus* and *Ruminococcus flavefaciens* were decreased (*p* < 0.001) by tannin treatments, and the magnitude of the decrease was greater with CHT5 treatment than with other treatments, but *Fibrobacter succinogenes* was increased (*p* < 0.001). Abundances of the proteolytic bacteria *Butyrivibrio fibrisolvens*, *Prevotella ruminicola*, and *Ruminobacer* *amylophilus* decreased (*p* < 0.001) in response to tannin treatments. Compared with the control, the CHQ2 and CHQ5 treatments did not affect *R*. *amylophilus* numbers.

## 4. Discussion

### 4.1. Fermentation Characteristics of Alfalfa Silage

In the present study, the addition of tannins, irrespective of type and level, improved the ensiling characteristics of alfalfa silage, as evidenced by reductions in DML, pH, acetic, and butyric acid concentrations, and NH_3_-N to TN ratios. Previous studies conducted by Li et al. [6] and Peng et al. [24] have shown similar results after tannin treatment on legume forages at ensiling. They attributed the improved fermentation efficiency to the antimicrobial properties of tannins that inhibited the undesirable microorganisms from breaking down fermentation substrates, such as proteins and carbohydrates, into silage acids, ethanol, and carbon dioxide. The growth of LAB was also inhibited by tannin treatments, as seen from the results of lactic acid and LAB, and the inhibitory effect was in a CHT dose-dependent manner. This finding suggested that HT has a greater ability to interfere with bacterial flora than CT during ensiling. Although the lactic acid fermentation was depressed in silages produced using tannin treatments, the pH for tannin treatments was lower than that for the control, and CHQ2 silage had the lowest pH value. In general, the extent of pH decline in silage is positively correlated to lactic acid yields during ensiling while negatively correlated to the concentration of proteolysis products such as NH_3_-N [9]. Thus, the variables in lactic acid and proteolysis product concentrations between these silages might contribute to the mixed pH values. Furthermore, pH is a simple indicator of the extent of silage fermentation, and pH with a range of 4.3 to 5.0 is acceptable for legume silages [9]. However, only the pH of CHQ2 and CHQ5 treatments was in this range. Together with lower NH_3_-N to TN ratios for CHQ2 and CHQ5 treatments than CHT2 and CHT5 treatments, this revealed that the combination of CHT and QT further improved silage fermentation quality than CHT alone.

The main concern for alfalfa silage quality is extensive proteolysis due to plant proteases and microbial activities during ensiling, which would produce large amounts of NPN compounds, consequently lowering nitrogen utilization and increasing nitrogen excretion into the environment by ruminants. The ensiling process resulted in extensive proteolysis of alfalfa, reflected by the changes in the proportion of PA and PB fractions before and after ensiling of alfalfa (265 vs. 703 g kg^−x^ CP, 685 vs. 235 g kg^−C^ CP, respectively). Additionally, the PA proportion (703 g kg^−C^ DM) for the control silage was greater than that reported by Contreras-Govea et al. [25]. This should be due to the lower DM concentration of alfalfa silage in the present study than that in their study because PA proportion is negatively correlated to DM concentration of silage [8]. Despite the similar CP concentration among all treatments, the decreased PA and increased PB proportions by tannin treatments clearly confirmed that tannin addition reduced the proteolysis of silage when compared with the control. Tannins have the ability to bind protein forming chemically stable complexes at pH 3.5 to 7 [26]; thus, protein in the complexes was kept from being degraded by plant proteases and microbial activities during ensiling. Regarding the composition of the PB fraction, the increase in PB_2_ proportions by tannin treatments was expected. The PB_2_ fraction generally degraded slowly in the rumen, leading to a high proportion of true protein flow to the intestine [6], which benefits protein utilization by ruminants. Importantly, we found that the average magnitudes of PA decrease and PB increase were greater in silages produced using combinations of CHT and QT than CHT alone, which indicated that combinations of HT and CT were more effective in reducing silage proteolysis than HT alone. Plant proteases are regarded as the primary actor in proteolysis during ensiling and initiate true protein degradation forming peptides and free amino acids, which are subsequently converted into NH_3_ by microbial activities. Therefore, it might be due to the fact that CT has a greater affinity to proteins and proteinases than HT [4], leading to a greater reduction in proteolysis by the combination of CHT and QT than CHT alone. Tannins can bind to fiber and thus increased the concentration of NDF.

### 4.2. Gas and CH_4_ Production and Rumen Microbiota

The decreased gas production for tannin treatments compared with the control indicated that tannin addition limited ruminal carbohydrate fermentation because rumen gases are primarily produced along with VFA formation from carbohydrate digestion. The decreased total VFA concentrations for tannin treatments partly supported this finding. Notwithstanding that ruminal CH_4_ is synthesized by methanogenic archaea using H_2_ and CO_2_ as substrates [27], it is accepted that tannins can reduce ruminal CH_4_ production in two ways: (1) inhibiting the activity of several rumen microbes associated with CH_4_ production; and (2) reducing carbohydrate digestion via forming stable complexes with carbohydrates [28].

Regarding the target rumen microbes, all tannin treatments directly decreased the abundance of total methanogens, which is the most closely associated with CH_4_ formation [29]. However, the magnitude of total methanogens population reductions in different tannin treatments did not match the reduction in CH_4_ production. This indicated that other microbial activities that reduce H_2_ production or provide an alternative pathway for H_2_ sink have been performed during in vitro fermentation. As important candidates for CH_4_ production, protozoa can be symbiotically associated with methanogens and serve abundant H_2_ for CH_4_ synthesis with their hydrogenosomes [30]. Previous studies have reported that removal of rumen protozoa leads to a 9–37% decrease in CH_4_ production [31,32]. Thus, the decreased protozoa populations for tannin treatments only at the high level might partly cause the CH_4_ variables. Previous studies have reported that ruminal anaerobic fungi have a similar H_2_-producing system as protozoa and interact with methanogens through interspecies H_2_ transfer [33,34]. Thus, the decreased anaerobic fungi populations due to tannin treatments might have lowered the H_2_ available for methanogens and further inhibited ruminal methanogenesis. Similarly, Jayanegara et al. [11] and Khiaosa-ard et al. [35] found a reduction in the abundance of ruminal anaerobic fungi accompanied by CH_4_ mitigation in the fermenter that received tannins.

The major cellulolytic bacteria *R. albus* and *R. flavefaciens* are representative H_2_ producers and have been shown to produce CH_4_ when in co-culture with methanogens [36,37]. In the present study, all tannin treatments decreased the abundance of *R. albus* and *R. flavefaciens* and, thus, lowered the H_2_ available for methanogens. In addition, we found that *R. albus* was more sensitive to tannins than *R. flavefaciens* according to the magnitude of the reduction of these two bacteria population by tannin treatments. This finding is in agreement with Wang et al. [38], who showed that the abundance of *R. albus* decreased by phlorotannin at 24 h incubation compared with the control, but that of *R. flavefaciens* was not affected by phlorotannin over the 24 h incubation. However, in contrast, Khiaosa-ard et al. [35] reported that *R. flavefaciens* was more susceptible to grape seed tannins than *R. albus*. Interestingly, unlike *R. albus* and *R. flavefaciens*, the growth of major cellulolytic bacteria *F. succinogenes* was enhanced by tannin treatments. Given that the cell wall of bacteria is the primary site where tannins exert inhibitory action [39], the differences in the cell wall structure between *F. succinogenes* (Gram-negative bacteria) and the two cellulolytic ruminococcus (Gram-positive bacteria) may result in the variable sensitivities to tannins. Furthermore, *F. succinogenes* does not produce H_2_ and thus is less sensitive to H_2_ accumulation due to loss of methanogens than *R. albus* and *R. flavefaciens* [40]. Therefore, a compensatory increase in *F. succinogenes* populations may occur after a decrease in the two cellulolytic ruminococcus populations.

The CHT treatment alone possessed a greater inhibitory effect on the anaerobic fungi *R. albus* and *R. flavefaciens* than the combination of CHT and QT, as revealed by the average magnitude of microbial population reduction by these two types of tannin treatments. However, the average CH_4_ production of CHQ2 and CHQ5 treatments was lower than that of CHT2 and CHT5 treatments. This suggested that QT in the combinations plays a significant role in further reducing CH_4_ production. Generally, HT can be catabolized to acetic and butyric acids with 3-hydroxy-5-oxohexanoate pathways in the rumen [41]; thus, the inhibitory effect of CHT on CH_4_ formation may become weak due to its hydrolyzation as incubation time increases. On the other hand, CT is difficult to be degraded in the rumen and has a greater affinity to carbohydrates than HT [4]. Collectively, this indicated that QT may contribute more to CH_4_ mitigation than CHT in the later stages of in vitro incubation, and the combination of HT and CT is more effective on CH_4_ mitigation than HT alone.

### 4.3. Rumen Fermentation and Rumen Microbes

The in vitro ruminal pH of the treatments varied between 6.75 and 6.85, which were within a normal range of ruminal pH from 5.5 to 7.0. Confirming the results of previous studies [28,42], tannin treatments decreased total VFA concentrations compared with the control. The decreased VFA production corresponded to the decreased DM and NDF degradation in this study. A recent meta-analysis showed that inhibition of methanogenesis shifts the rumen fermentation toward propionic acid production in batch cultures, which is an alternative pathway for consuming reducing equivalents and H_2_ utilization in the rumen [43]. In addition, we observed that the propionic acid molar proportion increased along with the decrease in CH_4_ production by tannin treatments. It should be noted that the microbial species targeted herein account for a fraction of the rumen microbes; thus, it is hard to explain the VFA results with the rumen microbes quantified. Considering that propionic acid is primarily produced through a succinate–propionate pathway in the rumen [29], the increased propionic acid production by tannin treatments may result from the increased *F. succinogenes* population, known as ruminal succinate producer. Regarding the host animal, an increase in propionic acid formation through chemical treatments is energetically beneficial because the gluconeogenesis of ruminants mainly depends on propionic acid supplementation [44].

A decrease in the concentration of NH_3_-N represents not only reduced protein degradation during rumen fermentation but improved nitrogen utilization by rumen microbes for MCP formation. Both cases were found in tannin treatments in the present study. The first case was reflected in the decreased CP degradation by tannin treatments compared with the control and supported by depressed molar proportions of iso-valeric and iso-butyric acids, end products of deamination of feed amino acids; the second case was confirmed by greater MCP concentrations in tannin treatments than the control. The increase in MCP formation was most likely due to the action of tannins, which slowed down the degradation of proteins and carbohydrates and thus provided a better synchronization of available nutrients [45]. Although the concentration of MCP was greater in CHT5 treatment than CHT2 treatment, CHQ5 treatment did not further increase MCP production compared with CHQ2 treatment. This may be due to the lowest nutrient degradation for CHQ5 treatment that appeared to decrease nitrogen and energy availability for rumen microbes, which restricted more MCP formation. It is known that the metabolizable protein (MP) flow to the intestine typically contains ruminally undegradable feed protein and MCP. The decreased CP degradation together with increased MCP by tannin treatments would be highly favorable to supply more MP to the host animal, which is an effective way to improve nitrogen utilization efficiency [46].

All tannin treatments suppressed DM, CP, and NDF degradation in the present study, which was in agreement with other studies that reported decreased nutrient degradation when tannins were added to feed in vivo [47]. Besides the protein-binding effects of tannins, the negative response of CP degradation to tannin treatments was due to the inhibition of the major proteolytic bacteria *B. fibrisolvens*, *P. ruminicola,* and *R. amylophilus*, which was reflected by the decreased proteinase activity in tannin treatments. Similarly, in addition to carbohydrate-binding effects, the decreased NDF degradation was related to the reduction in the abundance of the aforementioned cellulolytic microbes as well as the activity of cellobiase, xylanase, and pectinase by tannin treatments. The DM degradation was lower in CHQ2 and CHQ5 treatments than CHT2 and CHT5 treatments, although higher proteolytic and cellulolytic bacteria numbers were observed in CHQ2 and CHQ5 treatments than CHT2 and CHT5 treatments. This indicated that the further decreased DM degradation in CHQ2 and CHQ5 treatments is more likely due to the protein- and carbohydrate-binding capacity of tannins. As CP degradation was not affected and NDF degradation was decreased by the combination of CHT and QT than CHT alone (CHQ2 vs. CHT2 and CHQ5 vs. CHT5, respectively), the decreased DM degradation was most likely to be associated with carbohydrate fractions. Opposite to our expectation, the results indicated that tannins in this study had a greater affinity to carbohydrates than to protein. Furthermore, the results also confirmed that CT had a greater nutrient-binding capacity than HT.

## 5. Conclusions

Adding CHT to supply HT, alone or in combination with QT to supply CT, to alfalfa at ensiling at a low (20 g/kg DM) or high level (50 g/kg DM) reduced proteolysis, ruminal CH_4_ production, and NH_3_-N concentrations of alfalfa silage. Moreover, HT and CT in combination were more potent in modulating proteolysis and methanogenesis than HT only; however, the high level of inclusion will impair silage degradation and microbiota of the rumen. The results from this study revealed that a combination of HT and CT with complementary mechanisms at low levels can be a sustainable and effective strategy to improve the nitrogen utilization efficiency and CH_4_ mitigation of silage feed without adverse effects on ruminal fermentation patterns and microbiota. Further animal feeding studies are needed to validate our in vitro findings.

## Figures and Tables

**Table 1:** Chemical composition and microbial counts of fresh alfalfa.

Item ^a^	Alfalfa
DM (g kg^−1^)	235
pH	6.31
Buffer capacity (mEq kg^−1^ DM)	426
CP (g kg^−1^ DM)	233
PA (g kg^−1^ CP)	275
PB (g kg^−1^ CP)	680
PC (g kg^−1^ CP)	45
NDF (g kg^−1^ DM)	306
ADF (g kg^−1^ DM)	259
WSC (g kg^−1^ DM)	46.5
LAB (log_10_ cfu g^−1^ FM)	4.86
Yeasts (log_10_ cfu g^−1^ FM)	4.85

^a^ DM = dry matter; CP = crude protein; PA = non-protein nitrogen; PB = true protein; PC = undegradable protein; NDF = natural detergent fiber; ADF = acid detergent fiber; WSC = water-soluble carbohydrates; LAB = lactic acid bacteria; FM = fresh matter.

**Table 2:** Fermentation characteristics, microbial counts, and chemical composition of alfalfa silage treated without or with tannin additives.

Item ^A^	Treatments ^B^					SEM	*p*-Value
	Control	CHT2	CHT5	CHQ2	CHQ5		
Fermentation Characteristics							
DML (g kg^−1^)	96.2 ^a^	75.9 ^b^	74.1 ^bc^	63.7 ^d^	65.6 ^cd^	3.17	<0.001
pH	5.32 ^a^	5.13 ^b^	5.03 ^c^	4.92 ^d^	5.00 ^cd^	0.04	<0.001
Lactic acid (g kg^−1^ DM)	33.3 ^a^	31.3 ^b^	25.0 ^d^	33.6 ^a^	27.6 ^c^	0.91	<0.001
Acetic acid (g kg^−1^ DM)	18.6 ^a^	17.0 ^b^	13.7 ^d^	16.0 ^c^	14.5 ^d^	0.46	<0.001
Butyric acid (g kg^−1^ DM)	2.35 ^a^	0.79 ^b^	0.07 ^c^	0.09 ^c^	0.07 ^c^	0.24	<0.001
NH_3_-N (g kg^−1^ TN)	120 ^a^	88.9 ^b^	77.8 ^c^	69.7 ^d^	60.1 ^e^	5.56	<0.001
Microbial counts							
LAB (log_10_ cfu g^−1^ FM)	7.34 ^a^	6.84 ^bc^	5.88 ^d^	6.98 ^b^	6.70 ^c^	0.23	<0.001
Yeasts (log_10_ cfu g^−1^ FM)	4.26	4.26	4.23	4.25	4.24	0.10	0.856
Chemical composition							
DM (g kg^−1^ DM)	215 ^b^	226 ^a^	224 ^a^	229 ^a^	228 ^a^	1.52	0.004
CP (g kg^−1^ DM)	233	228	230	227	229	1.39	0.720
NDF (g kg^−1^ DM)	316 ^c^	325 ^b^	328 ^ab^	326 ^ab^	329 ^a^	1.23	<0.001
ADF (g kg^−1^ DM)	260	258	259	251	258	1.24	0.233

^A^ DML = dry matter loss; NH_3_-N = ammonia–nitrogen; LAB = lactic acid bacteria; DM = dry matter; CP = crude protein; NDF; neutral detergent fiber; ADF = acid detergent fiber. ^B^ Control = no tannins; CHT2 = 20 g kg^−1^ DM of chestnut tannin; CHT5 = 50 g kg^−1^ DM of chestnut tannin; CHQ2 = 10 g kg^−1^ DM of chestnut tannin + 10 g kg^−1^ DM of quebracho tannin; CHQ5 = 25 g kg^−1^ DM of chestnut tannin + 25 g kg^−1^ DM of quebracho tannin. Within a row, means without a common superscript letter (a–e) differ (*p* < 0.05).

**Table 3:** Ruminal fermentation characteristics and nutrient degradation in the in vitro fermentation cultures receiving untreated and tannin-treated alfalfa silage.

Item ^A^	Treatments ^B^					SEM	*p*-Value
	Control	CHT2	CHT5	CHQ2	CHQ5		
Fermentation characteristics							
pH	6.75	6.79	6.75	6.79	6.78	0.01	0.766
Total VFA (m*M*)	64.6 ^a^	59.5 ^b^	57.8 ^bc^	60.9 ^b^	55.3 ^c^	0.87	<0.001
VFA, mol 100 mol^−1^							
Acetic acid	68.9	68.8	68.4	68.3	68.7	0.09	0.143
Propionic acid	13.8 ^c^	15.3 ^b^	15.8 ^a^	15.9 ^a^	16.0 ^a^	0.22	<0.001
Iso-butyric acid	1.84 ^a^	1.53 ^b^	1.50 ^bc^	1.51 ^b^	1.35 ^c^	0.04	<0.001
Butyric acid	9.29	9.09	9.06	9.10	9.03	0.04	0.346
Iso-valeric acid	3.53 ^a^	3.09 ^b^	3.19 ^b^	3.17 ^b^	2.85 ^c^	0.06	<0.001
Valeric acid	2.11^a^	1.73 ^b^	1.75 ^b^	1.76 ^b^	1.70 ^b^	0.04	<0.001
Acetic acid/propionic acid	4.98 ^a^	4.46 ^b^	4.30 ^c^	4.31 ^c^	4.28 ^c^	0.07	<0.001
NH_3_-N (mg dL^−1^)	27.0 ^a^	23.7 ^b^	21.4 ^c^	22.4 ^bc^	20.1 ^d^	0.59	<0.001
MCP (mg dL^−1^)	25.8 ^c^	27.1 ^b^	28.5 ^a^	28.1 ^a^	28.2 ^a^	0.27	<0.001
Nutrient degradation							
DM (g kg^−1^)	636 ^a^	617 ^b^	586 ^c^	608 ^b^	568 ^d^	6.37	<0.001
CP (g kg^−1^)	861 ^a^	840 ^b^	832 ^cd^	838 ^bc^	829 ^d^	3.13	<0.001
NDF (g kg^−1^)	466 ^a^	444 ^b^	422 ^c^	435 ^b^	406 ^d^	5.75	<0.001

^A^ VFA = volatile fatty acids; NH_3_-N = ammonia–nitrogen; MCP = microbial protein; DM = dry matter; CP = crude protein; NDF = neutral detergent fiber. ^B^ Control = no tannins; CHT20 = 20 g kg^−1^ DM of chestnut tannin; CHT50 = 50 g kg^−1^ DM of chestnut tannin; CHQ2 = 10 g kg^−1^ DM of chestnut tannin + 10 g kg^−1^ DM of quebracho tannin; CHQ5 = 25 g kg^−1^ DM of chestnut tannin + 25 g kg^−1^ DM of quebracho tannin. Within a row, means without a common superscript letter (a–d) differ (*p* < 0.05).

**Table 4:** Ruminal enzyme activity and microbes in the in vitro fermentation cultures receiving untreated or tannin-treated alfalfa silage.

Item	Treatments ^A^					SEM	*p*-Value
	Control	CHT2	CHT5	CHQ2	CHQ5		
Enzyme activity ^B^							
Carboxymethyl-cellulase	0.624 ^a^	0.604 ^abc^	0.578 ^bc^	0.623 ^ab^	0.574 ^c^	0.01	0.011
Cellobiase	1.36 ^a^	1.22 ^b^	1.04 ^d^	1.17 ^bc^	1.09 ^cd^	0.03	<0.001
Xylanase	3.35 ^a^	2.49 ^c^	2.21 ^d^	2.96 ^b^	2.94 ^b^	0.11	<0.001
Pectinase	3.29 ^a^	2.55 ^c^	2.32 ^c^	2.96 ^b^	2.61 ^c^	0.10	0.001
α-amylase	12.3 ^a^	9.87 ^bc^	8.37 ^c^	11.5 ^ab^	11.6 ^ab^	0.41	<0.001
Protease	4.93 ^a^	4.48 ^b^	3.66 ^d^	4.60 ^b^	4.65 ^b^	0.12	<0.001
Microbes (copies mL^−1^)							
Total bacteria, ×10^11^	1.30	1.37	1.27	1.35	1.39	0.04	0.841
Total anaerobic fungi, ×10^7^	7.74 ^a^	5.94 ^c^	4.41 ^d^	6.63 ^b^	5.87 ^c^	0.29	<0.001
Total protozoa, ×10^6^	6.69 ^a^	6.63 ^a^	3.55 ^b^	6.68 ^a^	3.83 ^b^	0.39	<0.001
Total methanogens, ×10^8^	4.08 ^a^	3.31 ^b^	2.60 ^c^	3.29 ^b^	2.86 ^bc^	0.14	<0.001
*Rumincoccus albus*, ×10^8^	3.26 ^a^	2.17 ^c^	1.38 ^d^	2.92 ^b^	2.21 ^c^	0.18	<0.001
*Rumincoccus flavefaciens*, ×10^9^	1.81 ^a^	1.54 ^b^	1.25 ^d^	1.64 ^b^	1.41 ^c^	0.05	<0.001
*Fibrobacter succinogenes*, ×10^9^	3.69 ^c^	4.66 ^b^	4.44 ^b^	5.35 ^a^	5.19 ^a^	0.16	<0.001
*Butyrivibrio fibrisolvens*, ×10^7^	4.12 ^a^	2.87 ^c^	2.56 ^c^	3.68 ^b^	3.56 ^b^	0.16	<0.001
*Prevotella ruminicola*, ×10^10^	5.63 ^a^	3.67 ^d^	2.91^e^	4.70 ^c^	5.16 ^b^	0.27	<0.001
*Ruminobacer amylophilus*, ×10^7^	2.86 ^a^	2.44 ^b^	2.06 ^c^	2.86 ^a^	2.98 ^a^	0.10	<0.001

^A^ Control = no tannins; CHT2 = 20 g kg^−1^ DM of chestnut tannin; CHT5 = 50 g kg^−1^ DM of chestnut tannin; CHQ2 = 10 g kg^−1^ DM of chestnut tannin + 10 g kg^−1^ DM of quebracho tannin; CHQ5 = 25 g kg^−1^ DM of chestnut tannin + 25 g kg^−1^ DM of quebracho tannin. ^B^ Units of enzyme activity are carboxymethyl-cellulase (μmol glucose min^−1^ mL^−1^), cellobiase (μmol glucose min^−1^ mL^−1^), xylanase (μmol xylose min^−1^ mL^−1^), pectinase (μmol D-galactouronic acid min^−1^ mL^−1^), α-amylase (μmol glucose min^−1^ mL^−1^), and protease (μg hydrolyzed protein min^−1^ mL^−1^). Within a row, means without a common superscript letter (a–e) differ (*p* < 0.05).

## Data Availability

Data sharing not applicable.

## Supplementary Materials

The following are available online at https://www.mdpi.com/article/10.3390/ani11071967/s1, Table S1: Primers of microbes used for real-time PCR assay.
