# Supplementary material for: Effects of Hydrolysable Tannin with or without Condensed Tannin on Alfalfa Silage Fermentation Characteristics and In Vitro Ruminal Methane Production, Fermentation Patterns, and Microbiota
Source: Animals (Basel). 2021 Jun 30;11(7):1967. doi: 10.3390/ani11071967 (PMC8300162; doi:10.3390/ani11071967)
Supplement: Supplementary file 1 [file animals-11-01967-s001.zip › animals-1218465-supplementary.pdf]

Table S1

Primers of microbes used for real-time PCR assay.

Target species	Primer sequence (5’)	GeneBank accession no.	Annealing temperature (℃)	Size (bp)	Efficiency (%)
Total bacteria	F: CGGCAACGAGCGCAACCC<br>R: CCATTGTAGCACGTGTGTAGCC	AY548787.1	60	147	103.2
Total anaerobic fungi	F: GAGGAAGTAAAAGTCGTAACAAGGTTTC<br>R: CAAATTCACAAAGGGTAGGATGATT	GQ355327.1	57.5	120	90.2
Total protozoa	F: GCTTTCGWTGGTAGTGTATT<br>R: CTTGCCCTCYAATCGTWCT	HM212038.1	59	234	97.5
Total methanogens	F: TTCGGTGGATCDCARAGRGC<br>R: GBARGTCGWAWCCGTAGAATCC	GQ339873.1	60	160	102.5
Rumincoccus albus	F: CCCTAAAAGCAGTCTTAGTTCG<br>R: CCTCCTTGCGGTTAGAACA	CP002403.1	60	176	90.1
Rumincoccus flavefaciens	F: ATTGTCCCAGTTCAGATTGC<br>R: GGCGTCCTCATTGCTGTTAG	AB849343.1	60	173	90.8
Butyrivibrio fibrisolvens	F: ACCGCATAAGCGCACGGA<br>R: CGGGTCCATCTTGTACCGATAAAT	HQ404372.1	61	65	99.1
Fibrobacter succinogenes	F: GTTCGGAATTACTGGGCGTAAA<br>R: CGCCTGCCCCTGAACTATC	AB275512.1	61	121	94.8
Pevotella ruminicola	F: GAAAGTCGGATTAATGCTCTATGTTG<br>R: CATCCTATAGCGGTAAACCTTTGG	LT975683.1	58.5	74	94.6
Ruminobacer amylophilus	F: CTGGGGAGCTGCCTGAATG<br>R: GCATCTGAATGCGACTGGTTG	MH708240.1	60	102	93.7
